# Health or harm? A cohort study of the importance of job quality in extended workforce participation by older adults

**DOI:** 10.1186/s12889-016-3478-y

**Published:** 2016-08-25

**Authors:** Jennifer Welsh, Lyndall Strazdins, Sara Charlesworth, Carol T. Kulik, Peter Butterworth

**Affiliations:** 1 National Centre for Epidemiology and Population Health, Research School of Population Health, Australian National University, Building 62, Crn Mills and Eggleston Road, Canberra, ACT 2601 Australia; 2 School of Management, College of Business, RMIT University, 448 Swanston St, Melbourne, VIC 3000 Australia; 3 School of Management, University of South Australia, Elton Mayo Building, Corner of North Terrace and George Street, Adelaide, SA 5001 Australia; 4 Centre for Mental Health, Melbourne School of Population and Global Health; and Melbourne Institute of Applied Economic and Social Research, The University of Melbourne, Level 4, 207 Bouverie St, Parkville, VIC 3010 Australia

**Keywords:** Job quality, Employment, Retirement, Population ageing, Mental health, Physical activity, Functioning, Self-rated health

## Abstract

**Background:**

As people are living longer, they are being encouraged to work longer. While it is assumed that extended employment will be good for health, the evidence has been mixed. This study considers whether employment and job quality exert an influence on four indicators of health status in older workers.

**Methods:**

Data for this study came from 836 older workers (440 men and 396 women) aged 50–59 years at baseline who participated in the Household, Income and Labour Dynamics in Australia (HILDA) Survey. Using linear regression, we examine within-person change in self-rated, physical and mental health and one health behaviour (physical activity) at two time points over a nine year follow-up period.

**Results:**

There were minimal differences in the way health changed for older adults who continued working compared to those who retired voluntarily. However, when we decomposed employment in terms of job quality, health outcomes diverged. Compared to voluntary retirees, older workers who had worked in good quality jobs reported marginally better self-rated health (0.14,−0.02–0.29); but did not differ in their physical (2.31,−1.09–5.72) or mental health (0.51,−1.84–2.87). In contrast, older workers who held poor quality jobs for most of the follow-up period declined in their self-rated (−1.13,−0.28 − –0.02), physical (−4.90, 8.52– − 1.29) and mental health (−4.67, 7.69– − 1.66) relative to voluntary retirees. Older workers who held poor quality jobs for just some of the follow-up period did not differ from voluntary retirees in terms of their health. However there was evidence of a linear relationship between length of exposure to poor quality jobs and decline in health outcomes.

**Conclusion:**

Extended working lives mean that people will be ‘exposed’ to work for longer, and this exposure will occur at a life stage characterised by declining health for many. Our findings show that ensuring older workers have access to secure jobs which allow for control over work time, skill use and fair rewards will be essential if policy goals to boost participation and productivity, as well as reduce the health and care costs of the elderly, are to be met.

**Electronic supplementary material:**

The online version of this article (doi:10.1186/s12889-016-3478-y) contains supplementary material, which is available to authorized users.

## Background

All OECD countries face increasing health burdens related to non-communicable diseases. In 2012, 83 % of the burden of disease in high income countries was attributable to chronic health conditions such as cardiovascular disease, cancers, depression and diabetes, a number which has increased by 3 % since 2000 [1]. These countries are also dealing with the challenges posed by population ageing. The old age dependency ratio (the number of individuals aged 65 years or older relative to those within prime working age, 20–64 years) is projected to increase across the OECD from 24 % in 2005 to 52 % in 2050, meaning that there will be less than two workers for every person aged 65 years or older [2]. While the scale of the problem varies between countries, these changes are projected to have profound social and economic ramifications across the OECD. The most pressing of these ramifications will be a shrinking labour force (i.e. smaller pool of tax payers) coupled with increased demand for public spending on age-related pensions, health and aged care. Action is needed across multiple fronts and boosting participation rates of those aged 55–64 years is widely seen as an important part of the solution [3]. This paper investigates how four key health indicators are affected by policies designed to boost the number of older workers.

Currently, about two-thirds of people aged between 50 to 64 years participate in paid work in developed economies. Although cross-national variation is large [3], mobilising this untapped pool of older workers is expected to produce multiple and complementary benefits. A greater number of older adults in employment can increase both labour supply and consumption, benefiting the economy and increasing the tax base [3]. Greater participation by older workers decreases reliance on state-funded pensions and may improve financial preparedness for retirement [4]. On a societal level, extended participation recognises the value of older adults, the skills they possess and their capacity to contribute in meaningful and productive ways [5]. Further, it is often assumed that extended employment delivers health benefits [6] although there is currently no analysis of the health effects of extended participation for older adults.

While health is integrally connected to whether older workers stay in the labour market or leave early, the extent to which working into later life will deliver net benefits to health is not clear. Not all jobs improve health [7, 8]. It is therefore possible that health benefits or costs of extended employment depend on a range of factors, especially job quality and working conditions. Extended working lives mean that people will be ‘exposed’ to work for longer, and this exposure will occur at a life stage that is often characterised by declining health.

So far the evidence for the health benefits or costs of staying employed for older workers has been mixed and inconsistent [9, 10]. Much of the research is confounded by the ‘healthy worker effect’, whereby older workers in good health remain in the labour market and those with poor health leave early [11]. Once prior health is considered the evidence becomes more consistent. Studies have shown that compared to sustained employment, retirement is associated with short term improvements in self-rated health [12] and decreased morbidity from chronic health conditions [13], but has no effects on mortality [14] or physical or mental health [15]. Continued employment appears to support incidental physical activity in older workers but compromises leisure-time physical activity [16]. Thus, considering whether retirement decisions are driven by health (a form of involuntary retirement) or not (voluntary retirement) is vital [9].

A second weakness in the evidence on the health benefits or costs of extended employment relates to job quality. Previous research has underscored how the work-health relationship depends on the characteristics of people’s jobs and the demands or supports in the work environment. The concept of job quality has emerged as way of understanding the co-occurrence of job characteristics that support or undermine the well-being of workers, including their health [17, 18]. There are varying definitions of job quality and it is useful to distinguish between employment quality and job quality. Employment quality is focused more on aspects relating to the employment contract, such as wages and working hours, and job quality relating more to the way work is organized, such as workloads and job control [19]. In our study we define job quality in terms of the psychosocial characteristics known to be important for health and wellbeing: the use of worker’s skills, job control, job security, and the balance between effort and reward.

Numerous studies attest to the way jobs can have a direct impact on workers’ health, although few focus on older workers. For example, work demands and job control have been identified as key social determinants of cardiovascular disease and stress [20] and adverse working conditions and job hazards have been associated with poorer mental health [21]. There is also evidence that psychosocial stress related to the characteristics of employment can erode health more generally [8] and compromise health behaviours [22]. Further, recent studies indicate that poor quality jobs can be more detrimental to mental health than unemployment [7, 23]. In contrast, good quality jobs, marked by characteristics such as job control, employee flexibility and low levels of strain, support better health outcomes [8, 24] and have been associated with more health-promoting behaviours such as physical activity [25]. Mature age workers experience higher rates of chronic disease than their younger counterparts (see for example [26]), and therefore may be more vulnerable to work-related health risks.

Where job quality is integrated into research on older workers, it is largely as a predictor of retirement, not of the health of those who stay employed. European studies have revealed that job characteristics and psychosocial strain are related to retirement and retirement intentions e.g. [27, 28]. There may be different triggers to early retirement for different groups of workers. For example job autonomy and psychological stress are more likely to predict exit among professional workers whereas physical demands and psychological stress are more important for blue-collar workers [29]. However, there currently is no evidence on the associations between job quality and health changes in older workers. Our study integrates these lines of evidence to propose that job quality will determine if continuing to work while older delivers benefits or costs to health.

## Method

### Data and sample

Data for this study came from the Household, Income and Labour Dynamics of Australia (HILDA) Survey. The HILDA Survey is a nationally representative, household-based panel survey of Australian adults aged 15 years and older. Data is collected annually, primarily through interviews; however a small amount of information (relating to more sensitive topics, including health) is collected using a mailed back Self-Completion Questionnaire (SCQ), which is returned by approximately 90 % of the responding sample. In wave one, 13,969 respondents from 11,693 households were interviewed, representing a 61 % household response rate and 92.3 % individual response rate [30]. Response rates for the subsequent waves are approximately 90 %, which is comparable to other similar household based panel surveys [31]. Comparisons with census data reveal that the sample is broadly representative of the adult Australian population [32].

**Fig. 1:**
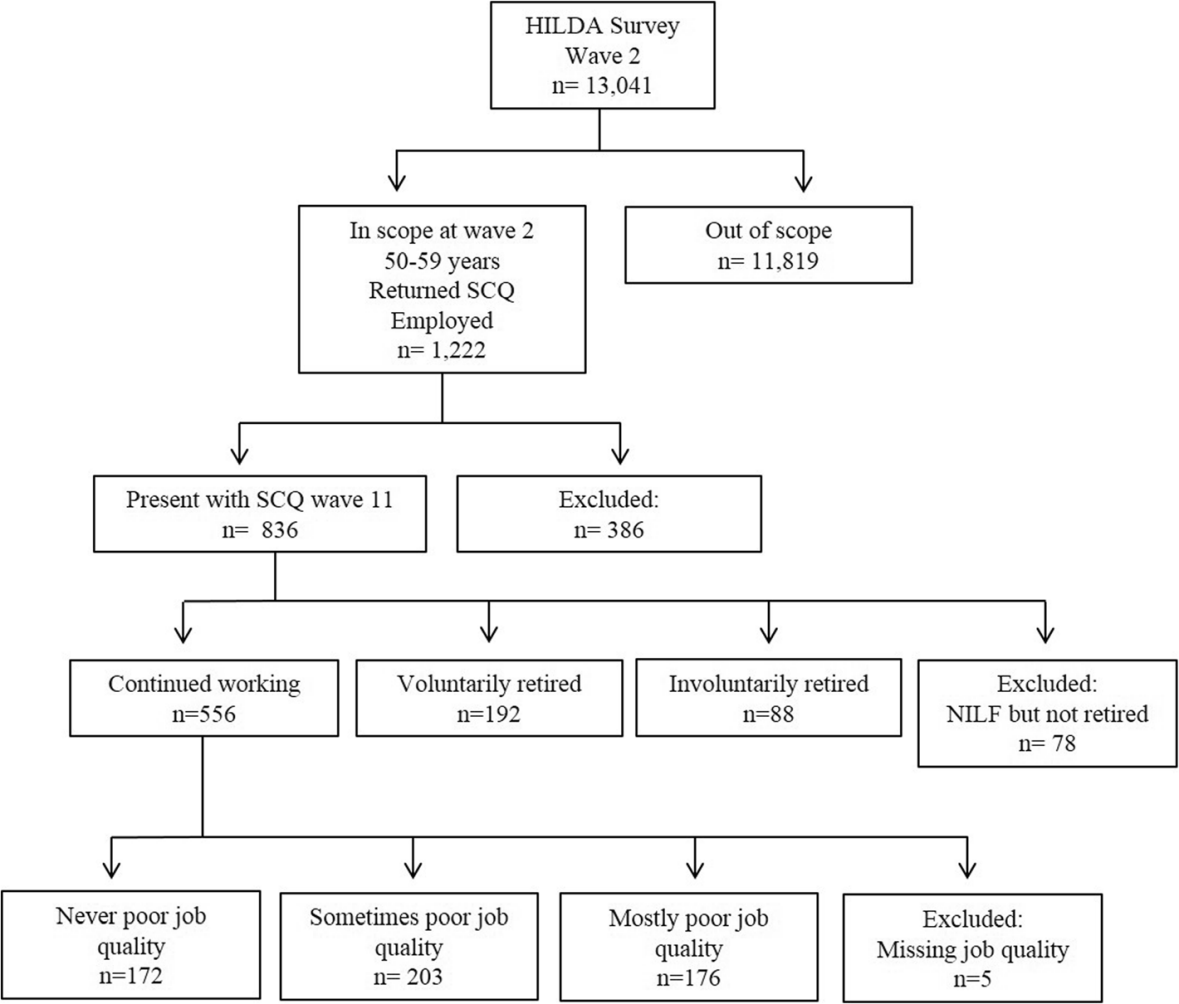
Participation flow and study numbers

This study used nine waves of data. Our baseline was wave 2 as a number of important items changed between waves 1 and 2, and wave 11 was the end point (where we made use of a retirement module). There were 13,041 individuals from 7,245 households who responded in wave 2. Respondents were included in this study if they satisfied the following criteria: aged 50–59 in 2002 (the year of wave 2 data collection); employed in wave 2; and, were present and returned their SCQ in both waves 2 and 11 of the survey. The participation flow diagram is presented in Fig. 1.

### Measures

Respondents were considered *employed* if they worked in a job, business or farm in the past seven days, or if they usually met those criteria but did not work in the past week because of holidays, sickness, or other reason. Respondents were considered *retired* if they did not participate in paid work in the previous seven days, and cited “retirement/ voluntarily inactive” as the main reason for doing so. All retired respondents were also asked in wave 11 if their decision to retire was “something that you wanted to do or something you felt you were forced or pressured to do”, which allowed us to classify people into two retirement groups. If they were “pressured/forced to” or “part wanted, part pressured/forced” they were considered *involuntary retirees*, whereas those who reported that they “wanted to” were termed *voluntary retirees*. People who were employed at baseline and in wave 11 were categorised as *continuing workers*.

We measured three health outcomes and one health behaviour. Physical functioning and poor mental health were measured with two subscales of the SF-36, which has been validated in the HILDA Survey [33]. The *physical functioning* subscale measures limitations in everyday activities over the previous four week period. Respondents are asked to rate (on a scale from 1 “yes, limited a lot” to 3 “no, not limited at all”) if they are limited by their health in a list of 10 activities, ranging from ‘vigorous activities’ (e.g. running) to simple everyday activities such as ‘bathing and dressing yourself’. The *mental health* subscale measures how often in the past four weeks respondents experienced positive (e.g. feeling ‘happy’) and negative (e.g. being ‘nervous’) emotions, symptomatic of common mental disorders [34]. Responses are recorded on a six-point scale from “all of the time” to “none of the time” to reflect mental distress and symptoms associated with common mental disorders (For information on the psychometric properties of these scales in the HILDA survey, see [33]).

*Self*-*rated health* was measured with one-item which asked respondents to rate their health ‘in general’ on a five point scale from “poor” to “excellent”. This measure has been shown to be a good indicator of health and mortality [35]. *Physical activity* was measured with a single item: ‘in general, how often do you participate in moderate or physical activity for at least 30 min’. Participants responded on a six-point scale which ranged from “not at all” to “everyday”.

For all four health measures, we calculated a *change in health* score between 2 time points over the nine year follow-up period by subtracting wave 11 scores from the baseline (wave 2) score. Negative scores represented deterioration in health, positive scores represented improvements to health and a score of 0 represented no change.

*Job quality* was measured with a validated measure of psychosocial job quality developed from the HILDA Survey [36]. This measures four components of job quality: skill use (two-items), job control (three-items), job security (three-items) and effort-reward fairness (one-item) (full item list is in Table 1). Following approaches used by others [8, 37], we averaged the items for each component and then dichotomised them at the point closest to the bottom quartile. A composite measure was created by summing the number of times respondents were in the bottom quartile for each component; scores were 0 (never in the bottom quartile), 1 (lowest quartile on one component) or 2 (lowest quartile on two, three or four components).

**Table 1:** HILDA Job Quality Components and Items

Component	Item
Skill use	My job requires learning new skills
	I use my skills in my current job
Job control	I have the freedom to decide how I do my work
	I have a lot of say about what happens at my job
	I have the freedom to decide when I do my job
Job security	I have a secure future in my job
	The company I work for will be in business in the next 5 years
	I worry about the future of my job (reverse coded)
Effort-reward fairness	I get paid fairly for the things I do

Notes: Responses for each item range from 0 “strongly disagree to 6 “strongly agree”

We measured *cumulative job quality* by calculating the number of years respondents reported two or more poor conditions (a score of 2 in the measure described above) over the follow-up period (waves 3 to 11). This was calculated using a ratio measure because almost 50 % of respondents had missing data on at least one-item across the follow-up (see missing data below). The measure was created by calculating the number of years the respondent reported a poor quality job divided by the number of waves for which the respondent had a score. Scores ranged from 0 % (never reported a poor quality job) to 100 % (always reported a poor quality job) and were then categorised into three groups: never (0 %), sometimes (1–49 %) and mostly (50–100 %) worked in a poor quality job. The validity of this measure was checked against a count measure of poor job quality summing the number of waves in which respondents reported having two or more poor conditions (and was not calculated for those with missing data). Scores on this measure ranged from never, sometimes (poor job for 1–4 years) and mostly (poor job for 5 or more years).

### Control variables

We also measured sociodemographic and health characteristics at baseline to include in our models as covariates. *Age* was measured continuously in years; participant *gender* and whether or not the respondent reported a *health condition* were coded as dichotomous categories.

### Additional control variables

Sensitivity analyses (described below) tested whether controlling for a larger number of baseline variables altered our results. As set out in Table 2, the additional covariates included: household post tax *income*, measured in quintiles; *education*; *marital status*; *employment status*, *occupational grouping*; and *contract status*. We also controlled for *adult care* (respondents who reported anything greater than 0 h per week caring for an adult due to illness, disability or old age), *grandchild care* (anything greater than 0 h per week caring for other people’s children on a regular and unpaid basis) and two important health behaviours: s*moking status* and *alcohol consumption* (abstainers: never or no longer drink; light drinkers: drinks alcohol ‘rarely’ to ‘1–2 times per week’; and drinkers: drinks ‘3 or times per week’ to every day).

**Table 2:** Baseline characteristics of the sample by retirement groups

	Continued working		Voluntarily Retired		Involuntarily Retired		Total	
	*N* = 556 (66.5 %)		*N* =192 (30.0 %)		*N* = 88 (10.5 %)		*N* = 836 (100 %)	
	N	Row %	N	Row %	N	Row %	N	Col %
Age (mean)	53.2		55.3		55.1			
Gender								
Men	310	70.5	84	19.1	46	10.5	440	56.3
Women	246	62.1	108	27.3	42	10.6	396	43.7
Health condition								
No	477	66.7	169	23.6	69	9.7	715	85.7
Yes	79	65.3	23	19.0	19	15.7	121	14.3
Household income (mean AUD/quintile)								
Poorest: $15,096	96	60.0	37	23.1	27	16.9	160	17.2
2: $31,015	107	64.9	40	24.2	18	10.9	165	19.4
3: $46,203	122	73.1	33	19.8	12	7.2	167	21.6
4: $64,084	120	70.2	38	22.2	13	7.6	171	21.6
Richest: $111,051	111	64.2	44	25.4	18	10.4	173	20.2
Education level								
High school or below	232	62.5	90	24.3	49	13.2	371	41.9
Certificate or diploma	174	70.2	50	20.2	24	9.7	248	30.9
Bachelor or higher	150	69.1	52	24.0	15	6.9	217	27.2
Marital Status								
Married/de facto	434	65.9	159	24.1	66	10.0	659	78.0
Single	122	68.9	33	18.6	22	12.4	177	22.0
Employment status								
Full-time (≥35 h)	419	69.7	124	20.6	58	9.7	601	75.5
Part-time (<35 h)	137	58.3	68	28.9	30	12.8	235	24.5
Occupation								
White collar	253	66.1	95	24.8	35	9.1	383	45.6
Blue collar	134	68.7	36	18.5	25	12.8	195	24.0
Pink collar	169	65.5	61	23.6	28	10.9	258	30.5
Contract status								
Ongoing/ fixed term	331	65.5	121	24.0	53	10.5	505	86.4
Casual	52	56.5	23	25.0	17	18.5	92	13.6
Adult care giving								
No	459	68.1	152	22.6	63	9.4	674	88.2
Yes	62	56.4	29	26.4	19	17.3	110	11.8
Grand child care								
No	464	67.1	157	22.7	71	10.3	692	89.2
Yes	56	61.5	24	26.4	11	12.1	91	10.9
Smoking status								
Never a smoker	296	67.9	102	23.4	38	8.7	436	53.8
Past smoker	180	65.7	61	22.3	33	12.0	274	32.4
Smoker	76	64.4	25	21.2	17	14.4	118	13.9
Alcohol consumption								
Abstainer	60	65.2	20	21.7	12	13.0	92	10.9
Light	277	69.4	77	19.3	45	11.3	399	49.6
Drinkers	218	63.4	95	27.6	31	9.0	344	39.5

Note: Percentages may not sum to 100 due to rounding

### Statistical approach

The first set of analyses considered health changes between the two time points (wave 2 and wave 11) among older adults comparing those who remained employed and those who retired voluntarily and involuntarily. The second set decomposed the group of continuing workers to consider the extent their job quality determined health costs or benefits of sustained employment.

Linear regression models were used to test whether group membership predicted change in health scores. Each health outcome was represented as a change score (the difference between the wave 11 and wave 2 scores). For each health outcome, a series of models were used to: (i) evaluate the significance of the differences observed in health between respondents classified as voluntary retirees and the other groups of interest; and (ii) estimate the change observed in health within each group. Both sets of analyses were adjusted for potential confounders. For each outcome measure, we report crude change scores from simple models including only employment status, and adjusted change scores, derived from models which included the coefficients from the full multivariate model (estimates were derived using the ‘margins’ command within Stata). To aid comparison across the different outcome measures, the final adjusted change scores were expressed as a percentage of the potential range of each scale (dividing the estimated change score for each group by the original scale range). Retirement was added into the models using two categories, with voluntary retirees as the reference group. Separate analyses were computed for each health outcome, and following the method presented by others [38], relative and overall change scores were adjusted for age, gender, presence of a health condition and baseline health score. The equation used to generate these models is given below:

$$ {Y}_j={\beta}_0+{\beta}_1{X}_1+{\beta}_2{X}_2+{\beta}_{Ci}{X}_{Ci}+\in $$

where Y is change for each of the health measures

*j* (physical functioning, mental health, self-rated health or physical activity)

β_0_ is the intercept

X_1_ and *X*_2_ are the key (dummy coded) covariates representing involuntary retirement and continuing employment

β_1_ and β_2_ are the corresponding coefficients,

X_*Ci*_ represents the additional covariates included in the model (e.g., age, gender, health condition),

with β_*Ci*_ the corresponding coefficients.

These models were repeated and extended by the further decomposition of the continuing employment category *X*_2_:

$$ {Y}_j={\beta}_0+{\beta}_1{X}_1+{\beta}_3{X}_3+{\beta}_4{X}_4+{\beta}_5{X}_5+{\beta}_{Ci}{X}_{Ci}+\in $$

to differentiate those respondents who never had a poor quality job (X_3_), who in some waves had a poor quality job (X_4_), and those who had a poor quality job in most waves (X_5_).

The strength of this approach is that it estimates within-person changes between the two time points and allows for meaningful health comparisons between retirement and employment. Sensitivity analyses tested whether further adjustment for additional baseline sociodemographic and health behaviours characteristics (see additional control variables above) altered the relationship substantively. We then examined the social distribution of poor quality jobs to identify which groups of older workers were most at risk. Cramér’s V was calculated to test the overall associations between job quality and sociodemographic variables.

Missing data for those who met the study criteria were low (less than 5 %), with the exception of job quality. While missing data on the individual job quality items was low (approximately 10 %), almost 50 % of continuing workers had missing data on at least one job quality item at some point in the study, and had a missing score for our composite job quality measure as a result. Approximately 90 % of missing data on this job quality measure was ‘valid’, that is, respondents were not in the labour force at that point in the study; the remaining sources of missing were due to non-participation in a particular wave (generally less than 1 % per wave), failing to return the SCQ (1–2 % per wave) or item refusal (<1 % per wave). To overcome this, a ratio measure of job quality was used in our main analyses (see measures above). However, a series of sensitivity analyses tested whether excluding those with missing data altered the relationship. All other missing data was excluded using listwise deletion.

### Statement on ethics

The HILDA Survey is administered by the Melbourne Institute and was approved by the Melbourne University Ethics committee. Respondents provided consent to take part in the study and parental consent was obtained for respondents aged less than 18 years. Our secondary analysis was approved by the Human Research Ethics Committee at the University of South Australia (project ID: 0000032602) and the Australian National University Human Ethics Committee (project ID: 2014/117).

## Results

There were 923 respondents who met the study inclusion criteria. However, 78 respondents were excluded because they reported something other than retirement as the main reason for not being employed (such as home duties, own or others’ illness/ disability or doing unpaid work) and nine respondents were excluded because they did not provide a reason for their retirement. This resulted in a final sample of 836 respondents, of whom 556 (66.5 %) were classified as continuing workers, 192 (22.9 %) as voluntary retirees and 88 (10.5 %) as involuntary retirees. Of the continuing workers, 172 (31.2 %) never reported a poor quality job, 203 (36.8 %) reported a poor quality job in some of the follow-up period and 176 (31.9 %) mostly reported a poor job quality in that period. Five (<1 %) continuing workers never reported job quality information and were dropped from the second set of analyses. The overall sample had a mean age of 53.9 year at baseline. However, continuing workers were on average two years younger than those who retired during the follow-up period. Table 2 describes the sample characteristics.

### Health changes over nine years: Comparing employment and retirement

Table 3 presents the results from the first analysis testing the association of (voluntary and involuntary) retirement and employment, with health and physical activity. Between the two time points in the follow-up period, self-rated health and physical functioning declined, mental health improved and there were no changes evident in reported physical activity. As expected, involuntary retirees reported the poorest health and the lowest levels of physical activity at baseline, and showed the greatest deterioration in self-rated, mental and physical health over the follow-up period. However, their rates of physical activity increased after retirement. Continuing workers and voluntary retirees reported similar levels of health at baseline and in crude change between the two time points; however unlike both groups of retirees, continuing workers reported a decrease in their physical activity over time.

**Table 3:** Mean health at baseline, crude mean change and adjusted difference in change in health

Group Membership	Baseline mean	Crude change scores	Model coefficient (adjusted)^ab^	Adjusted change scores (%)^ac^
	(95 % CI)	(95 % CI)	(95 % CI)	(95 % CI)
Self-Rated health (scale 1–5) *N* = 813				
Involuntarily retired	3.10 (2.92–3.28)	−0.38 (−0.56– − 0.20)	−0.37 (−0.57– − 0.17) ***	−10.80 (−14.20– − 7.40)***
Voluntarily retired	3.49 (3.38–3.60)	−0.17 (−0.29– − 0.05)	0.00 (ref)	−3.40 (−5.40– − 1.20)**
Continued working	3.60 (3.53–3.67)	−0.21 (−0.27– − 0.14)	−0.01 (−0.14–0.11)	−3.60 (−4.80– − 2.40)***
Physical Functioning (scale 0–100) *N* = 808				
Involuntarily retired	75.26 (70.16–80.36)	−7.06 (−11.81– − 2.32)	−9.24 (13.97– − 4.52)***	−11.93 (−16.06– − 7.80)***
Voluntarily retired	85.89 (83.38–88.40)	−3.48 (−6.16– − 0.81)	0.00 (ref)	−2.69 (−5.00– − 0.37)*
Continued working	87.27 (85.92–88.62)	−3.75 (−5.31– − 2.19)	−0.58 (−3.39–2.24)	−3.26 (−4.73– − 1.80)***
Mental Health (scale 0–100) *N* = 834				
Involuntarily retired	76.47 (72.97–79.97)	−0.38 (−3.43–2.68)	−4.92 (−8.22– − 1.62)**	−1.60 (−4.52–1.32)
Voluntarily retired	78.65 (76.58–80.71)	3.57 (1.58–5.55)	0.00 (ref)	3.33 (1.59–5.06)***
Continued working	78.99 (77.77–80.22)	1.38 (0.15–2.61)	−1.67 (−3.79–0.46)	1.66 (0.57–2.75)**
Physical Activity (scale 0–6) *N* = 833				
Involuntarily retired	3.22 (2.86–3.57)	0.49 (0.10–0.88)	0.12 (−0.25–0.49)	4.17 (−1.17–9.67)
Voluntarily retired	3.79 (3.58–4.00)	0.08 (−0.13–0.30)	0.00 (ref)	2.33 (−0.83–5.50)
Continued working	3.76 (3.63–3.88)	−0.10 (−0.23–0.03)	−0.22 (−0.45–0.01)^	−1.50 (−3.33–0.50)

Notes: ^*p* < 0.1, *p < 0.05,**p < 0.01, ***p < 0.001. ^a^Adjustments made for the following baseline characteristics: age, sex, health condition and health score. ^b^Significance refers to the difference between the group and the reference category (voluntary retirees). ^c^Significance refers to whether there was a change in the group’s health or physical activity. On all scales higher scores represent better health/ more physical activity

**Fig. 2:**
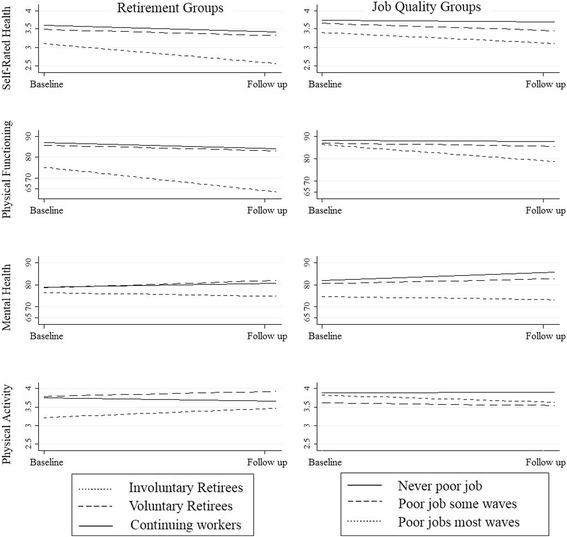
Baseline and adjusted follow up health and physical activity scores for each of the retirement and employment groups. Notes: Baseline scores are unadjusted; follow-up scores are adjusted for age, sex, health condition and health score. Adjusted estimates are calculated using the equations presented in the methods section

Linear regression models were used to predict difference between the three groups in their change in health and physical activity between two time points. Figure 2 presents the adjusted change scores for the three groups. The results for the three health outcomes were consistent for voluntary retirees and continuing workers: these two groups of respondents reported similar, small deteriorations in their self-rated and physical health, and small improvements in their mental health. The models showed no evidence of significant difference for self-rated health (*p* = 0.825), physical functioning (*p* = 0.687) or mental health (*p* = 0.123). In comparison, involuntary retirees’ reported significantly greater deterioration in their self-rated health (*p* < 0.001), physical functioning (*p* < 0.001) and mental health (*p* = 0.003) relative to voluntary retirees. Whereas voluntary retirees reported a 3.40 % (−5.40– − 1.20) decrease in their self-rated health and a 2.69 % (−5.00– − 0.37) decrease in their physical functioning, involuntary retirees reported a 9.00 % (−0.71– − 0.37) and an 11.93 % (−16.06– − 7.80) decrease respectively. Strikingly, involuntary retirees reported a (nonsignificant) 1.60 % (−4.52–1.32) decrease in their mental health score over time whereas voluntary retirees reported a 3.33 % (1.59–5.06) improvement in mental health.

Changes in physical activity showed a different pattern to the other outcomes. After adjustment, there was no significant difference between voluntary and involuntary retirees in frequency of physical activity from baseline to the end of the follow-up (*p* = 0.533). There was a trend among continuing workers (compared to voluntary retirees) for reduced levels of physical activity over time (*p* = 0.056). In comparison to voluntary retirees who reported a small but non-significant increase of 2.33 % in physical activity from baseline to follow-up, continuing workers reported a small but non-significant decrease of 1.50 %.

### Health changes, employment and job quality

A second set of analyses decomposed the continuing workers into three separate categories based upon job quality, to investigate how this influenced the association between employment and change in health between the two time periods. The first column of Table 4 shows how baseline health varied as a function of job quality. Those continuing workers who had mostly worked in poor quality jobs showed poorer self-rated and mental health, and lower levels of physical functioning at baseline compared with those who had never or only sometimes worked in poor quality jobs.

**Table 4:** Crude mean change and adjusted mean difference in change for all retirement groups

Group Membership	Baseline mean	Crude change scores	Model coefficient (adjusted)^ab^	Adjusted change scores (%)^ac^
	(95 % CI)	(95 % CI)	(95 % CI)	(95 % CI)
Self-Rated Health (scale 1–5) *N* = 808				
Involuntary retirement	3.10 (2.92–3.28)	−0.38 (−0.56– − 0.20)	−0.38 (−0.58– − 0.18)***	−11.00 (−14.20– − 7.60)***
Voluntary retirement	3.49 (3.38–3.60)	−0.17 (−0.29– − 0.05)	0.00 (ref)	−3.40 (−5.60– − 1.20)**
Never poor job	3.73 (3.61–3.85)	−0.12 (−0.23– − 0.01)	0.14 (−0.02–0.29)^	−0.60 (−2.80–1.40)
Poor job some waves	3.66 (3.54–3.77)	−0.25 (−0.36– − 0.14)	−0.03 (−0.18–0.12)	−4.00 (−6.00– − 2.00)***
Poor jobs most waves	3.41 (3.27–3.54)	−0.23 (−0.34– − 0.12)	−0.13 (−0.28–0.02)^	−6.00 (−8.00– − 3.80)***
Physical Functioning (scale 0–100) *N* = 803				
Involuntary retirement	75.26 (70.16–80.36)	−7.06 (−11.81– − 2.32)	−9.25 (−13.97–4.53)***	−12.02 (16.15– − 7.89)***
Voluntary retirement	85.89 (83.38–88.40)	−3.48 (−6.16– − 0.81)	0.00 (ref)	−2.77 (−5.08– − 0.46)**
Never poor job	88.38 (85.96–90.79)	−1.64 (−4.56–1.28)	2.31 (−1.09–5.72)	−0.45 (−2.91–2.00)
Poor job some waves	87.06 (84.76–89.37)	−1.98 (−4.24–0.29)	1.39 (−1.77–4.55)	−1.38 (−3.38–0.63)
Poor jobs most waves	86.42 (84.05–88.79)	−7.48 (−10.35– − 4.62)	−4.90 (−8.52– − 1.29)***	−7.67 (−10.43– − 4.92)***
Mental Health (scale 0–100) *N* = 829				
Involuntary retirement	76.47 (72.97–79.97)	−0.38 (−3.43–2.68)	−4.97 (−8.28– − 1.66)***	−1.65 (−4.58–1.28)
Voluntary retirement	78.65 (76.58–80.71)	3.57 (1.58–5.55)	0.00 (ref)	3.32 (1.58–5.06)***
Never poor job	81.91 (80.03–83.78)	2.10 (0.29–3.91)	0.51 (−1.84–2.87)	3.83 (2.28–5.38)***
Poor job some waves	80.54 (78.69–82.39)	0.99 (−0.74–2.27)	−1.17 (−3.54–1.19)	2.15 (0.61–3.69)***
Poor jobs most waves	74.65 (72.16–77.15)	0.78 (−2.00–3.55)	−4.67 (−7.69– − 1.66)***	−1.35 (−3.66–0.96)
Physical Activity (scale 0–6) *N* = 828				
Involuntary retirement	3.22 (2.86–3.57)	0.49 (0.10–0.88)	0.12 (−0.25–0.49)	4.17 (−1.17–9.67)
Voluntary retirement	3.79 (3.58–4.00)	0.08 (−0.13–0.30)	0.00 (ref)	2.17 (−1.00–0.53)
Never poor job	3.89 (3.67–4.11)	−0.08 (−0.29–0.14)	−0.13 (−0.40–0.15)	0.17 (−3.00–3.17)
Poor job some waves	3.61 (3.40–3.82)	−0.01 (−0.23–0.20)	−0.20 (−0.48–0.07)	−1.17 (−4.33–2.00)
Poor jobs most waves	3.82 (3.59–4.06)	−0.24 (0.48–0.01)	−0.32 (−0.61– − 0.03)*	−3.17 (−6.83–0.50)

Notes: ^*p* < 0.1, *p < 0.05,**p < 0.01, ***p < 0.001. ^a^Adjustments made for baseline characteristics: age, sex, health condition and health score. ^b^Significance refers to the difference between the group and the reference category (voluntary retirees). ^c^Significance refers to whether there was a change in the group’s health or physical activity. On all scales higher scores represent better health/ more physical activity

There were no statistically significant differences for changes in self-rated health between voluntary retirees and any of the job quality groups. Relative to voluntary retirees, workers who had never worked in a poor quality job reported marginally less deterioration in their self-rated health (*p* = 0.084) and workers who mostly worked in a poor quality job reported marginally more deterioration (*p* = 0.096). Compared to voluntary retirees whose self-rated health decreased by 3.40 % (−5.60– − 1.20), workers who had never worked in a poor quality job reported a decrease of just 0.60 % (−2.80–1.40) and those who mostly worked in poor quality jobs reported a decrease of 6.00 % (−8.00– − 3.80). There was no difference evident between voluntary retirees and workers who had only sometimes worked in poor quality jobs (*p* = 0.691).

The quality of employment was related to change in mental health, physical functioning and physical activity among continuing workers, generating distinctive changes in health compared with voluntary retirees. Older adults who mostly worked in a poor quality job reported greater deterioration over time in their physical functioning (*p* = 0.008), mental health (*p* = 0.002) and physical activity (*p* = 0.029) compared to voluntary retirees. Voluntary retirees reported a deterioration of 2.77 % (−5.08– − 0.46) in their physical functioning compared to a 7.67 % (−10.43– − 4.92) deterioration for workers who mostly worked in a poor quality job. Voluntary retirees reported a 3.32 % (1.58–5.06) improvement in their mental health, whereas older workers who mostly had a poor quality job reported a deterioration of 1.35 % (−3.66–0.96). Similarly, voluntary retirees reported a 2.17 % (−1.00– − 0.53) increase in physical activity, but workers who mostly worked in a poor quality job reported a 3.17 % (−6.83–0.50) decrease. In contrast, there were no significant differences between voluntary retirees and those who had never or only sometimes reported working in poor quality jobs. Nonetheless, point estimates provided evidence of a linear trend between the length of exposure to poor quality employment and the likelihood of poorer health outcomes. The predicted health outcomes by job quality are plotted in Fig. 2.

### Sociodemographic patterning of job quality

Our results indicate that the health benefits or costs of sustained employment are dependent upon job quality. We now describe the social patterning of job quality, to identify which older workers may be most at risk. As shown in Table 5, Cramer’s V estimates revealed relatively weak associations between categories of job quality and sociodemographic variables. Where associations were evidence, the patterning revealed that poor job quality was aligned with social disadvantage. Older men were more likely than women to work in the best or worst quality jobs, with older women more likely to sometimes work in a poor quality job over this period of follow up. Working in a relatively good quality job was linked with higher education attainment; 39.8 % of those with lower levels of education reported poor job quality for most of the follow-up compared to 20.7 % of those with tertiary education. Among older workers, those who were married were more likely to report never working in a poor quality job (33.0 %) compared to single older workers (24.8 %), as were full-time compared to part-time workers (33.9 % compared to 23.0 %). Casual workers (50.0 %) compared to ongoing or fixed-term employed (28.8 %) workers were also more likely to report a poor quality job for most of the follow-up period. White collar workers were less likely to report poor job quality for most of the follow-up period; just 24.3 % white collar workers reported that their job was poor quality for most of the waves compared to 30.4 % of pink collar and almost half (48.5 %) of blue collar workers. There were only small differences by adult or grandchild care responsibilities, and almost no difference by health status. There was, however, a clear gradient in income and job quality; 42.1 % of those in the poorest households reported mostly or always having a poor quality job, compared to just 15.3 % of those in the richest.

**Table 5:** Sociodemographic profile of those who experienced poor job quality for none, some or most of their follow-up for those who continued working

	Reported poor quality job during follow-up						Total	Cramér’ssr
	Never		Some		Most		*N* = 551	V
	*N* = 172		*N* = 203		*N* = 176		(100 %)	
	N	Row %	N	Row %	N	Row %	N	
Gender								0.138
Men	105	33.9	96	31.0	109	35.2	310	
Women	67	27.8	107	44.4	67	27.8	241	
Education level								0.152
High school or below	53	22.9	86	37.2	92	39.8	231	
Certificate or diploma	51	30.0	66	38.8	53	31.2	170	
Bachelor or higher	68	45.3	51	34.0	31	20.7	150	
Marital Status								0.089
Married or de facto	142	33.0	159	37.0	129	30.0	430	
Single	30	24.8	44	36.4	47	38.8	121	
Employment status								0.105
Full-time	141	33.9	150	36.1	125	30.1	416	
Part-time	31	23.0	53	39.3	51	37.8	135	
Contract Status								0.160
Ongoing or fixed term	108	32.7	127	38.5	95	28.8	330	
Casual	10	19.2	16	30.8	26	50.0	52	
Occupation								0.163
White collar	98	39.0	92	36.7	61	24.3	251	
Blue collar	29	22.0	39	29.6	64	48.5	132	
Pink collar	45	26.8	72	42.9	51	30.4	168	
Adult-care giving								0.069
No	150	32.9	165	36.2	141	30.9	456	
Yes	14	23.0	26	42.6	21	34.4	61	
Grandchild care								0.084
No	148	32.2	162	35.2	150	32.6	460	
Yes	15	26.8	27	48.2	14	25.0	56	
Health condition								0.017
No	146	30.9	174	36.9	152	32.2	472	
Yes	26	32.9	29	36.7	24	30.4	79	
Household income (quintiles)								0.177
Poorest	6	22.1	12	35.8	18	42.1	36	
2	17	22.4	33	41.1	34	36.5	84	
3	26	26.1	35	34.5	32	39.5	93	
4	42	35.3	55	37.0	56	27.7	153	
Richest	81	48.7	68	36.0	36	15.3	185	

Notes: Percentages may not sum to 100 due to rounding

### Sensitivity analyses

We tested the robustness of our results with two sets of sensitivity analyses. First, we included further covariates into our linear regression models to test if the observed patterns of health changes could be explained by a more comprehensive list of sociodemographic or health behaviour characteristics measured at baseline. The results (not shown here but available online in Additional file 1: Table S1) for the models which tested employment and job quality groupings revealed that the pattern and direction of the estimates for the predicted change in health outcomes and physical activity were largely unchanged (see Additional file 1: Tables S1 and S2). We also tested our models which decomposed employment by job quality using a count (not ratio) measure of job quality to consider if the choice of approach to handle missing data altered the results. Results of these models were also unchanged from analyses using the reported ratio measure (see Additional file 1: Table S3).

## Discussion

This paper examines the health effects of extended labour force participation, using a nationally representative sample of older Australian adults. Better understanding the nature of the relationship between health and employment into older age is vital as policy efforts to increase the pool of older workers are scaling up, responding to the demographic shifts and economic challenges of population ageing. While rarely discussed, the health outcomes of these policies will decide the extent that the anticipated benefits for individuals, workplaces and societies are realised. Unintended negative health consequences could compromise population health and undermine productivity, thereby increasing government spending on health and age-related services [39].

Our first set of analyses addressed a key conceptual and methodological problem: viewing retirees as a unitary group in terms of their health. Separating voluntary from involuntary retirees revealed distinctive health trajectories. Those who left the workforce involuntarily showed the poorest self-rated, mental and physical health at baseline and the sharpest declines in their health, a finding which accords well with previous research [14, 15]. By modelling involuntary retirement separately, we control for this health selection effect and minimise the potential for misinterpretation of the relationship between health and continued labour force participation relative to (voluntary) retirement. Furthermore, we then show that retirement, when voluntary, is not accompanied by health decline; our second key finding.

Voluntary retirees and continuing workers were found to have similar levels of health at the beginning of the study, and similar changes to their physical, self-rated and mental health over the follow-up period [15]. Both groups reported only modest declines in their self-rated health and physical functioning, and small improvements in their mental health. There was some evidence of a slight decline in physical activity linked to extended participation: while the difference between voluntary retirees and extended workers was only marginal, this finding suggests a potential trade-off between extended participation and physical activity, with long term implications for health [40].

In the second stage of our analysis, we investigated if the health effects of extended participation depended on the quality of the jobs held by older workers. By examining job quality, we consider the diversity of labour market experiences, which may generate a variety of health outcomes. Consistent with previous research [7, 8], our results revealed a distribution of health outcomes by job quality, which was itself socially patterned. Older workers who never reported a poor quality job bucked typical age-related trends in their health outcomes and reported no significant deteriorations to their health. While the changes they experienced to their physical functioning, mental health or physical activity health did not differ significantly from voluntary retirees, they did report modest, favourable, changes to their self-rated health. In contrast, older workers who continued to participate in jobs of poor quality reported significantly larger declines in physical functioning, mental health and physical activity levels relative to those who voluntarily retired. Significant health costs were only present in those who reported a poor quality job for most of the follow-up period, although there was evidence that health outcomes declined with longer exposure to poor quality employment.

### Limitations

There are several limitations of our study. The first is that we examined changes over two time points in a nine year period, not health trajectories using data from all nine waves. Health changes, especially in terms of chronic disease, are likely to take several years to be discernible, hence our analysis across a nine year span. Using more data points and modelling changes year by year might provide a deeper understanding of the dynamics of employment-related health trajectories in older workers. We did not model whether the association between job quality and health outcomes differed for those in part-time employment, or by occupation or industry. Controlling for these characteristics at the study baseline did not substantially alter the pattern of results. However it is possible that changes to these factors over the follow-up period are important for the association between employment and health, particularly for physical activity.

There are also limitations related to our measures. All of our measures were self-reported and two of our outcome measures (self-reported health and physical activity) were single items. Our single-item measure of physical activity also does not allow us to distinguish incidental activity from leisure time activity but future research should examine these associations using more measures of health and examine the ways in which these relationships differ for different types of workers.

Our final limitation relates to our measure of job quality. Our measure has shown to be a good indicator of a number of key aspects of psychosocial work environment, such as job control and effort reward fairness. Nevertheless, it does not measure other features of the work environment also crucial for health [18, 41] and is thus likely to provide a conservative estimate of the job quality-health relationship. Future research would benefit from using an expanded measure, that includes employment preferences, workloads, and physically demanding work, as well as considering whether some aspects of job quality are more important than others for supporting optimal health among older workers.

## Conclusion

While previous research has shown how job quality shapes early retirement intentions, our study reveals that job quality will also determine how the health of older workers is shaped and distributed when labour force participation is extended. On the one hand, extended employment in a high quality job could protect the health of older workers, slowing age-related declines in physical health and supporting greater age-related improvements to mental health. On the other hand, employment in poor quality jobs may erode good health, amplifying age-related declines in physical health and blocking access to age-related improvements to mental health. Our analysis reveals that both outcomes are possible consequences of increasing the participation rates of older workers, and that these very different outcomes are driven by job quality.

Our analysis of the sociodemographic distribution of job quality indicates that, at least in the Australian context, a relatively large number of older workers may be vulnerable; more than two-thirds of our sample reported poor quality jobs at some point during study period. Furthermore, the social patterning of job quality followed traditional axes of disadvantage: women, as well as single, less educated workers and people living in households with lower incomes were less likely to report being in optimal quality jobs, as were part-time, less skilled and casually employed workers. Poor quality employment could therefore compound social disadvantage, raising the possibility that policies to extend employment could exacerbate health inequities, unless they are paired with policies to address job quality. Furthermore, failure to address employment-related health declines has the potential to undermine the long-term success of these policies by increasing the demand for health and age-related services. If the anticipated social, health and economic benefits of extended labour force participation are to be realised, it will be essential that older workers have access to good quality jobs. That is, secure jobs that deliver fair rewards, allow for use of workers’ skills and provides some measure of job control.

## Additional file

Additional file 1: Supplementary tables.doc–tables presenting the results of the analyses which are adjusted for a larger number of covariates (Tables S1 and S2) and adjusted for the count (rather than ratio) measure of job quality (Table S3). (DOCX 21 kb)

## Abbreviations

HILDA: Household, Income and Labour Dynamics in Australia Survey
NILF: Not in the Labour Force
OECD: Organisation for Economic Co-operation and Development
SCQ: Self-completion Questionnaire
