# Supplementary material for: Health or harm? A cohort study of the importance of job quality in extended workforce participation by older adults
Source: BMC Public Health. 2016 Aug 25;16(1):885. doi: 10.1186/s12889-016-3478-y (PMC5000457; doi:10.1186/s12889-016-3478-y)
Supplement: Additional file 1: — Supplementary tables.doc–tables presenting the results of the analyses which are adjusted for a larger number of covariates (Tables S1 and S2) and adjusted for the count (rather than ratio) measure of job quality (Table S3). (DOCX 21 kb) [file 12889_2016_3478_MOESM1_ESM.docx]

Table S1. Mean health at baseline, crude mean change and adjusted difference in change in health.

| Group Membership | Adjusted relative change ^#1^  (95%CI) | Adjusted % change ^#2^  (95%CI) |
| --- | --- | --- |
| Self-Rated health (scale 1-5) N = 813 | |  |
| Involuntarily retired | -0.33 (-0.54 – -0.11)** | -10.04 (-13.70 – -6.38)*** |
| Voluntarily retired | 0.00 (ref) | -3.51 (-5.77 – -1.24)** |
| Continued working | -0.01 (-0.14-0.12) | -3.67 (-4.90 – -2.44)*** |
|  |  |  |
| Physical Functioning (scale 0-100) N = 808 | |  |
| Involuntarily retired | -7.91(-12.89 – -2.94)** | -10.71 (-15.05 – -6.37)*** |
| Voluntarily retired | 0.00 (ref) | -2.80 (-5.24 – -0.36)* |
| Continued working | -0.49 (-3.44 – 2.45) | -3.29 (-4.83 – -1.76)*** |
|  |  |  |
| Mental Health (scale 0-100) N = 834 | |  |
| Involuntarily retired | -5.50 (-9.05 – -1.95)** | -1.92 (-5.03 – 1.19) |
| Voluntarily retired | 0.00 (ref) | 3.58 (1.76 – 5.40) *** |
| Continued working | -1.93 (-4.11 – 0.26) ^ | 1.66 (0.52 – 2.79)** |
|  |  |  |
| Physical Activity (scale 0-6) N = 833 | |  |
| Involuntarily retired | 0.14 (-0.27 – 0.54) | 0.20 (-0.15 – 0.56) |
| Voluntarily retired | 0.00 (ref) | 0.07 (-0.13 – 0.27) |
| Continued working | -0.16 (-0.39 – 0.08) | -0.09 (-0.21 – 0.03) |

Notes: ^ p<0.1, * p<0.05,** p<0.01, *** p < 0.001. ^#^Adjustments made for the following baseline characteristics: age, sex, health condition, health score, education, part-time employment, relationship status, adult and grandchild caregiving responsibilities, smoking status, alcohol consumption and income quintiles. ^1^ Significance refers to the difference between the group and the reference category (voluntary retirees). ^2^ Significance refers to whether there was a change in the group’s health or physical activity over the follow up period. On all scales higher scores represent better health/ more physical activity.

Table S2. Crude mean change and adjusted mean difference in change for all retirement groups

| Group Membership | Adjusted relative change^#^  (95%CI) | Adjusted % change ^#^  (95%CI) |
| --- | --- | --- |
| Self-Rated Health (scale 1-5) N=808 | |  |
| Involuntary retirement | -0.33 (-0.55 – -0.12)** | -10.13 (-13.80 – -6.45)*** |
| Voluntary retirement | 0.00 (ref) | -3.45 (-5.72 – -1.20)** |
| Never poor job | 0.10 (-0.06 – 0.26) | -1.49 (3.72 – 0.74) |
| Poor job some waves | -0.04 (-0.19 – 0.12) | -4.16 (-6.23 – -2.09)*** |
| Poor jobs most waves | -0.08 (-0.24 – 0.07) | -5.13 (-7.27 – -2.99)*** |
|  |  |  |
| Physical Functioning (scale 0-100) N=803 | |  |
| Involuntary retirement | -8.22 (-13.19 – -3.24)** | -10.98 (-15.32 – -6.64)*** |
| Voluntary retirement | 0.00 (ref) | -2.77 (-5.21 – -0.32)* |
| Never poor job | 1.79 (-1.88 – 5.46) | -0.98 (-3.66 – 1.71) |
| Poor job some waves | 1.33 (-2.02 – 4.68) | -1.43 (-3.58 – 0.71) |
| Poor jobs most waves | -4.50 (-8.27 – -0.72)** | -7.26 (-10.12 – -4.40)*** |
|  |  |  |
| Mental Health (scale 0-100) N=829 | |  |
| Involuntary retirement | -5.72 (-9.29 – -2.15)** | -2.11 (-5.24 – 1.01) |
| Voluntary retirement | 0.00 (ref) | 3.60 (1.78 – 5.43)*** |
| Never poor job | -0.27 (-2.68 – 2.13) | 3.33 (1.71 – 4.95)*** |
| Poor job some waves | -1.34 (-3.80 – 1.12) | 2.27 (0.63 – 3.90)** |
| Poor jobs most waves | -4.73 (-7.93 – -1.53)** | -1.13 (-3.61 – 1.36) |
|  |  |  |
| Physical Activity (scale 0-6) N=828 | |  |
| Involuntary retirement | 0.13 (-0.27 – 0.54) | 3.37 (-2.55 – 9.28) |
| Voluntary retirement | 0.00 (ref) | 1.12 (2.21 – 4.45) |
| Never poor job | -0.10 (-0.38 – 0.18) | -0.55 (-3.88 – 2.78) |
| Poor job some waves | -0.13 (-0.42 – 0.15) | -1.10 (-4.33 – 2.12) |
| Poor jobs most waves | -0.25 (-0.55 – 0.05) | -3.00 (-6.74 – 0.73) |

Notes: ^ p<0.1, * p<0.05,** p<0.01, *** p < 0.001. ^#^Adjustments made for the following baseline characteristics: age, sex, health condition, health score, education, part-time employment, relationship status, adult and grandchild caregiving responsibilities, smoking status, alcohol consumption and income quintiles. ^1^ Significance refers to the difference between the group and the reference category (voluntary retirees). ^2^ Significance refers to whether there was a change in the group’s health or physical activity over the follow up period. On all scales higher scores represent better health/ more physical activity.

Table S3. Crude mean change and adjusted mean difference in change for all retirement groups using the count measure of job quality.

| Group Membership | Adjusted relative change^#^  (95%CI) N | Adjusted % change ^#^  (95%CI) |
| --- | --- | --- |
| Self-Rated Health (scale 1-5) |  |  |
| Involuntary retirement | -0.38 (-0.58 – -0.18)*** | -0.55 (-0.72 – -0.38)*** |
| Voluntary retirement | 0.00 (ref) | -0.17 (-0.28 – -0.06)** |
| Never poor job | -0.24 (0.05 – 0.42) * | 0.07 (-0.07 – 0.21) |
| Poor job some waves | -0.02 (-0.20 – 0.15) | -0.19 (-0.32 – -0.06)** |
| Poor jobs most waves | -0.13 (-0.35 – 0.09) | -0.30 (-0.49 – -0.11) ** |
|  |  |  |
| Physical Functioning (scale 0-100) | |  |
| Involuntary retirement | -9.06 (-13.77 – -4.34)*** | -11.73 (-15.83 – -7.63)*** |
| Voluntary retirement | 0.00 (ref) | -2.67 (-4.98 – -0.37)* |
| Never poor job | 4.95 (1..45 – 8.46)** | 2.28 (-0.31 – 4.87)^ |
| Poor job some waves | 2.22 (-1.15 – 5.58) | -0.45 (-2.76 – 1.85) |
| Poor jobs most waves | -4.21 (-9.31 – 0.90) | -6.88 (-11.34 – -2.41) ** |
|  |  |  |
| Mental Health (scale 0-100) |  |  |
| Involuntary retirement | -4.89 (-8.20 – -1.58)** | -1.29 (-4.22 – 1.64) |
| Voluntary retirement | 0.00 (ref) | 3.60 (1.85 – 5.35)*** |
| Never poor job | 0.77 (-2.05 – 3.60) | 4.37 (2.26 – 6.49)*** |
| Poor job some waves | -0.82 (-3.45 – 1.80) | 2.78 (0.89 – 4.66)** |
| Poor jobs most waves | -4.69(-9.68 – 0.30) ^ | -1.09 (-5.62 – 3.44) |
|  |  |  |
| Physical Activity (scale 0-6) |  |  |
| Involuntary retirement | 0.14 (-0.24 – 0.51) | 0.29 (-0.04 – 0.61)^ |
| Voluntary retirement | 0.00 (ref) | 0.15 (-0.04 – 0.34) |
| Never poor job | -0.01 (-0.31 – 0.28) | 0.14 (-0.08 – 0.36) |
| Poor job some waves | -0.16 (-0.45 – 0.13) | -0.01 (-0.22 – 0.21) |
| Poor jobs most waves | -0.24 (-0.63 – 0.15) | -0.09 (-0.42 – 0.25) |

Notes: ^ p<0.1, * p<0.05,** p<0.01, *** p < 0.001. ^#^Adjustments made for baseline characteristics: age, sex, health condition and health score. ^1^ Significance refers to the difference between the group and the reference category (voluntary retirees). ^2^ Significance refers to whether there was a change in the group’s health or physical activity over the follow up period. On all scales higher scores represent better health/ more physical activity.
